# Supplementary material for: SlimVar for rapid in vivo single-molecule tracking of chromatin regulators in plants
Source: Nat Commun. 2025 Sep 1;16:8156. doi: 10.1038/s41467-025-63108-8 (PMC12402101; doi:10.1038/s41467-025-63108-8)
Supplement: Supplementary file 1 — Supplementary Information [file 41467_2025_63108_MOESM1_ESM.pdf]

SlimVar for rapid *in vivo* single-molecule tracking of chromatin regulators in plants

Payne-Dwyer *et al.*

Supplementary Table 1. Sample statistics used for quantitative SlimVar analysis. Numbers of nuclei and tracks quantified**.** Col*FRI* acquisitions were aggregated over all tested vernalisation conditions: NV = not vernalised; V2W = two weeks of cold; V6W = six weeks of cold; V6WT7 = six weeks of cold followed by one week of warm conditions; V6WT14 = six weeks of cold followed by two weeks of warm conditions.

**Line**	**Vernalisation/imaging condition**	**Independent growth cycles**	**Cells quantified**	**Nuclear tracks detected**
VIN3-SYFP2	NV	3	22	0
	V2W	4	71	1,158
	V6W	3	46	896
	V6WT7	3	23	0
VIN3-GFP	NV	3	24	0
	V2W	5	57	994
	V6W	4	83	1,485
	V6WT7	4	36	0
VRN5-EYFP	NV	4	50	1,039
	V2W	3	36	964
	V6W	4	63	1,703
	V6WT14	4	31	813
VRN5-mScarletI	NV	5	44	552
	V2W	6	29	426
	V6W	2	16	256
	V6WT14	3	29	365
*FLC-lacO/*LacI-YFP	NV	3	38	295
VRN5-mScarletI × *FLC-lacO/*LacI-YFP	NV	3	10	365 × 23
	V2W	2	28	1,703 × 156
	V6W	3	25	2,867 × 192
	V6WT14	2	18	1,416 × 74
Col*FRI*	All (green / 488 nm)	3	79	0
	All (yellow / 514 nm)	3	38	0
	All (red / 561 nm)	3	79	0

Supplementary Table 2. Average stoichiometry of VIN3-GFP, VIN3-SYFP2 and VRN5-YFP assemblies whose diffusivity matches that of *FLC* loci. The diffusivity-matched stoichiometry is distributed similarly to the population (VIN3-SYFP2 V6W, $N = 296$ tracks, $p = 0.08$), and similarly increases with vernalisation for both VIN3 (V2W to V6W, SYFP2: $N = 262$, $p = 1 \times 10^{-12}$; GFP: $N = 302$, $p = 2 \times 10^{-9}$) and VRN5 (NV to V6W+T14, $N = 318$, $p = 3 \times 10^{-5}$). VIN3-SYFP2 values are corrected for the fraction of endogenous VIN3 (Supplementary Fig. 7). NV = not vernalised; V2W = two weeks of cold; V6W = six weeks of cold; V6WT14 = six weeks of cold followed by two weeks of warm conditions. Source data are provided as a Source Data file.

**Line**	**Timepoint**	***FLC*-like tracks $D \approx D_{FLC}$**		**Stoichiometry (molecules)**	
		**Number, n**	**% of total (± s.e.m.)**	**Median**	**IQR**
VIN3-GFP	V2W	151	15.2 (1.2)	9.9	5.9–16.3
	V6W	262	17.6 (1.1)	15.1	9.0–26.0
**VIN3-SYFP2**	V2W	131	11.3 (1.0)	3.2	2.0–6.0
				6.4	3.9–11.9 *corrected*
	V6W	148	16.5 (1.4)	5.2	3.8–8.4
				10.4	7.6–16.8 *corrected*
VRN5-YFP	NV	192	18.4 (1.3)	11.3	7.3–23.4
	V2W	168	17.4 (1.3)	17.7	11.1–29.9
	V6W	355	20.8 (1.1)	18.1	11.5–30.2
	V6W+T14	159	19.6 (1.2)	20.4	12.7–31.0

Supplementary Table 3. Primers used in this study.

Primer name	Primer sequence 5' -> 3'
**Primers for cloning**	
Mega-pENTR VIN3-mTurq2 F	GCGGCAGCTTCAGCCGTGAGCAAGGGCGAGGAG
Mega-pENTR VIN3-mTurq2 R	GAGTTAATAAATAGACCGATTTCAAAAGCTTTTACTTGTACAGCTCGTCCATG
VRN5_mScarlet_T	GCATCCGCGGCAGCTTCAGCCGTGAGCAAGGGCGAGGCA
VRN5_mScarlet_B	GTTGAATCAATGGGAAGCTTGATTACTTGTACAGCTCGTCCATG
**qRT-PCR primers**	
FP-qPCR-F2	GGACGACGGCAACTACAAGA
FP-qPCR-R2	GTTGCCGTCCTCCTTGAAG
VIN3-qPCR-1F	TGCTTGTGGATCGTCTTGTCA
VIN3-qPCR-1R	TTCTCCAGCATCCGAGCAAG
VRN5_qPCR_F	GAGGCATTGTTAGTAGGCTTCC
VRN5_qPCR_R	CACGCAGCTGGAACTAAATCTC
FLC spliced F	AGCCAAGAAGACCGAACTCA
FLC spliced R	TTTGTCCAGCAGGTGACATC
UBC_qPCR_F	CTGCGACTCAGGGAATCTTCTAA
UBC_qPCR_R	TTGTGCCATTGAATTGAACCC
PP2A F2	ACTGCATCTAAAGACAGAGTTCC
PP2A R2	CCAAGCATGGCCGTATCATGT

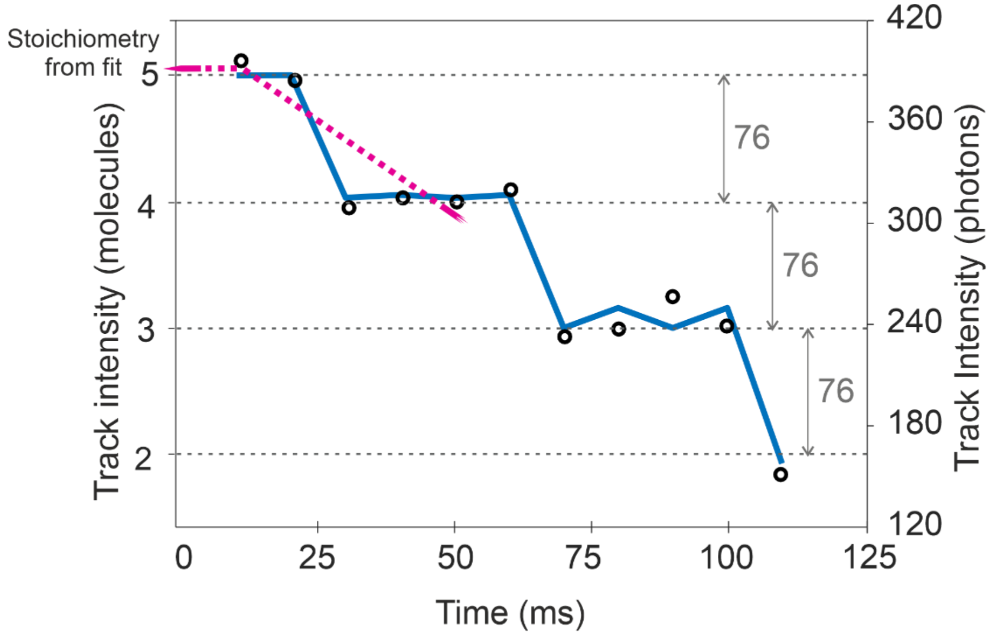

Supplementary Fig. 1. Determination of characteristic molecular brightness in live tissues from photobleaching. Intensity of foci (black circles) near the end of a single track in a post-vernalised VRN5-YFP image sequence, interpolated using a Chung-Kennedy edge-preserving filter (centred window of 3 points, blue line). With sufficient signal-to-noise, photobleaching enables counting of individual steps corresponding to the presence of individual labelled molecules in an assembly. The consistent photobleaching steps from fluorescent fusions provide enough resolution to count not only molecular steps in specific traces, but also to derive an ensemble estimate of the characteristic molecular brightness for working depths of 20 ± 10 µm. The steps yield an estimate of 76 ± 10 photons for the characteristic single-molecule brightness of the VRN5-YFP fusion *in planta*. Normalisation of the intensity trace by this value gives the absolute number of active fluorophore reporters over the entire course of photobleaching, which remains robust when individual steps would be missed or otherwise unidentifiable. A backward extrapolation of the first five points accounts for photobleaching and allows the track stoichiometry to be estimated with better precision than the individual foci. Source data are provided as a Source Data file

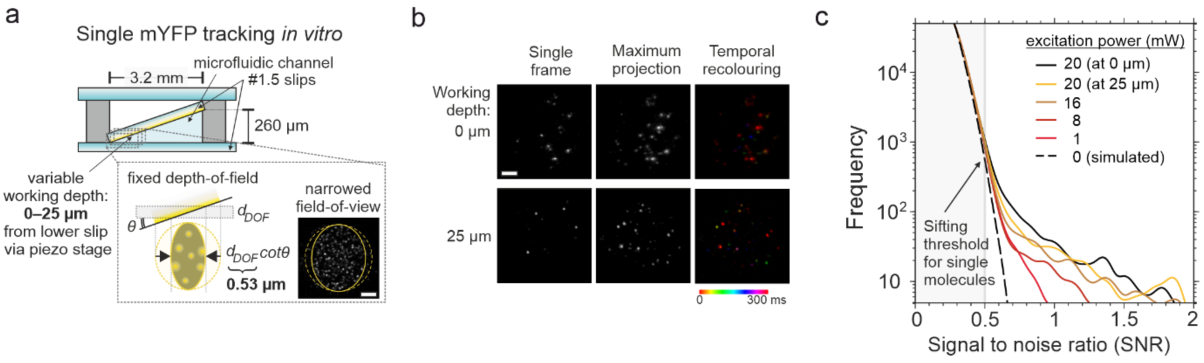

Supplementary Fig. 2. **SlimVar detects and quantifies single fluorescent proteins in foci above threshold levels of signal-to-noise.**

a) A channel with a tilted internal coverslip surface facilitated observation of single particles *in vitro* at a range of working depths. Purified YFP were tracked in 10 ms exposures, either at zero depth in epifluorescence mode or at a calibration depth of 25 µm in SlimVar mode. If the imaged surface is sufficiently tilted by an angle θ, the depth-of-field (d_{DOF}, the axial extent of the detection volume) limits the width of the region in focus to $d_{DOF} \cot\theta$ (inset scale bar 2 µm). At 25 µm working depth in SlimVar, we found the width to be 6,500 ± 300 nm, thus d_{DOF} = 530 ± 30 nm. At the emission wavelength of 528 nm, this result indicated an effective numerical aperture [1] of 1.38 ± 0.02 (from the nominal NA 1.49), representing better light collection than possible with water immersion (NA < 1.33).

b) YFP foci observed within a 300 ms window (20 mW excitation, 10 ms exposure per frame), showing similar performance in epifluorescence at zero depth, and in SlimVar at the calibration depth. The temporal recolouring indicates how foci appear (bind), diffuse in short, confined trajectories and disappear (photobleach or unbind); scale bar 2 µm.

c) The appropriate SNR threshold for sifting is the SNR above which single molecules are unlikely to be false positives, here at 0.5. We show the frequency vs. SNR of individual foci detected above a nominal cutoff of 0.2, in 10 fields-of-view at different excitation powers and working depths after SlimVar calibration: 20 mW power at the coverslip (zero working depth, solid black) or the calibration working depth of 25 µm at source excitation powers of 20 mW (yellow) and lower powers of 16 mW (brown), 8 mW (orange) and 1 mW (red). The distribution at zero power is derived from tracking simulations of Gaussian noise matched to the experimental dark detector noise (standard deviation of 2.3 photons/pixel) evaluated over three fields-of-view, equivalent to an average background of 15 photons/pixel.

Source data are provided as a Source Data file.

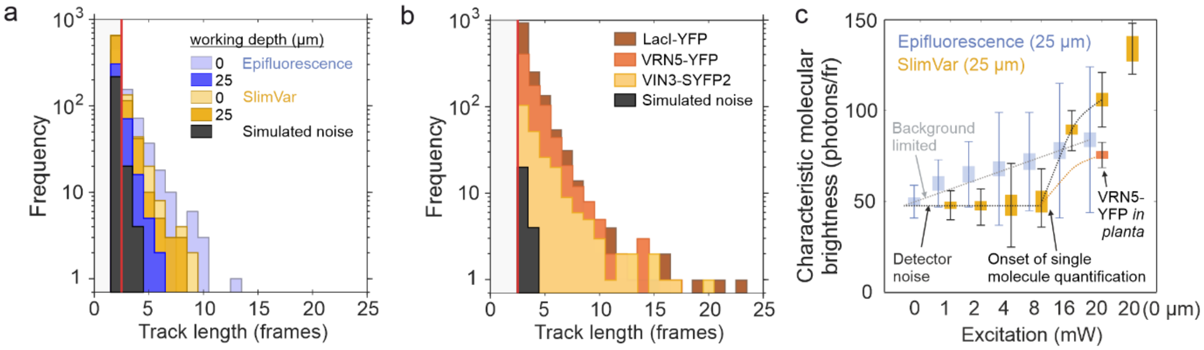

Supplementary Fig. 3. SlimVar detects and quantifies fluorescent proteins along tracks at a characteristic molecular brightness *in vitro* and in plants.

a) After sifting above the SNR threshold of 0.5, the tracks (same dataset as Supplementary Fig. 2) have a distribution of lengths (number of foci) which varies by modality and working depth. 'Epifluorescence' is performed at 0º beam incidence at 20 mW source power (blue), while 'SlimVar' refers to 60º beam incidence at 20 mW source power after the calibration protocol (yellow). The tracking length for epifluorescence deteriorates with increasing working depth (dark blue vs light blue), while those of SlimVar are less dependent on depth (dark yellow vs light yellow). The tracks of length 3 or more have a much lower overlap with false positives from noise (black), which motivates us to include a minimum track length of 3 in the sifting process.

b) Lengths of tracks detected in plants using SlimVar (LacI-YFP, brown; VRN5-YFP, orange; VIN3-SYFP2, yellow; noise, black). The track lengths for assemblies are correspondingly greater for species with higher mean stoichiometry and lower mean diffusivity.

c) The characteristic molecular brightness, estimated from the mean photons per frame in detected tracks, as a function of excitation power. The detector noise generates a floor value of 50 photons/frame, from the few false positives that inevitably pass the sifting step. For epifluorescence at depth (blue bars), the high background immediately causes the apparent value to increase with excitation power, but for isolated molecules, this is not well correlated with true positive events (panel d). For SlimVar at depth (yellow bars), the background is low and there is a clear onset above a critical power of ~8 mW (irradiance ~4 kW/cm^2) corresponding to the true number of photons detected from a single fluorescent protein. The value is highest at zero working depth (rightmost bar), decreasing slightly at the calibration working depth, and drops further due to scattering and aberrations in plants (orange), yet remains above the floor level. Source data are provided as a Source Data file.

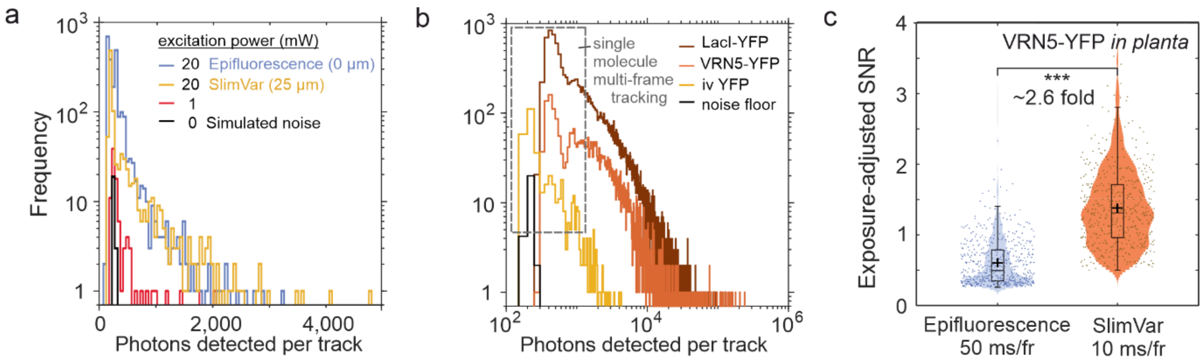

Supplementary Fig. 4. SlimVar detects and quantifies fluorescent proteins and assemblies at depth, both *in vitro* and in plants, with superior performance to epifluorescence.

a) The distribution of the total number of photons associated with each track (same single-molecule YFP dataset as Supplementary Fig. 2) for optimal epifluorescence at the surface and 20 mW source power (blue), optimal SlimVar at 25 µm working depth and 20 mW source power (yellow), compared with those of SlimVar at 1 mW source power (red), below the requirement for single molecule detection, and the simulated detector noise (black).

b) The total number of photons per track in vivo, plotted on a logarithmic scale (LacI-YFP, brown; VRN5-YFP, orange). In vitro controls are also shown for comparison (single molecule YFP, yellow; noise, black). The total increases for states with high stoichiometry and low diffusivity. Distinct peaks can be seen at low values, corresponding to single molecules integrated over a small discrete number of frames.

c) The signal-to-noise improvement in vivo due to the SlimVar modality for tracks of VRN5-YFP in plants. Epifluorescence is performed at a longer exposure of 50 ms/frame, while SlimVar is at the balanced mode (10 ms/frame). The SNR values shown account for the additional exposure time required by dividing the raw values for epifluorescence by a factor of $(50\text{ ms}/10\text{ ms})^{0.5}$. SlimVar detects additional tracks that are lower stoichiometry and higher diffusivity including single molecules, that for epifluorescence would have raw SNR below the sifting threshold of 0.5 and are therefore invisible. Even with epifluorescence showing this bias towards brighter particles, the mean signal-to-noise in SlimVar track is still at least 2.6× greater.

Source data are provided as a Source Data file.

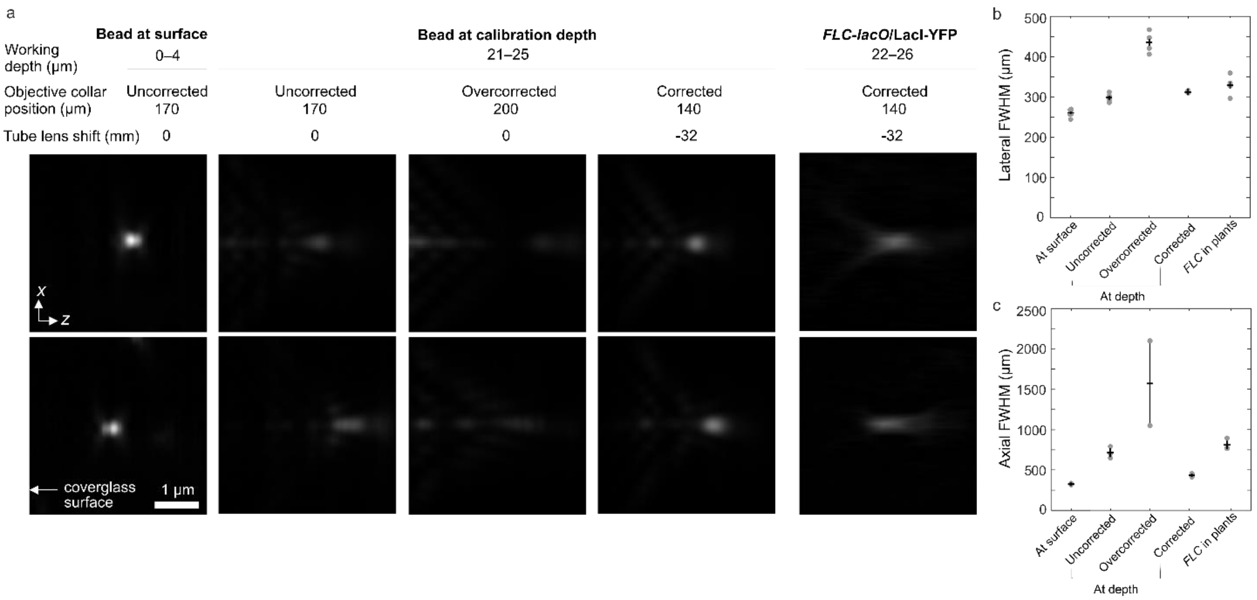

Supplementary Fig. 5. SlimVar sufficiently mitigates aberrations associated with high-numerical aperture imaging at a specified calibration depth.

a) Individual PSFs shown in axial cross section, acquired as volume stacks from either subdiffractive fluorescent beads (24 nm diameter FluoSpheres) suspended in 1 wt.% agarose at 488 nm wavelength excitation at 20 nm *z*-step, or LacI-YFP foci in root tip nuclei with 514 nm excitation at 250 nm step. Scale bar 1 µm. All PSFs are spatially upsampled to 20 nm (linear interpolation), background-subtracted and normalised to the same total pixel intensity. Top and bottom rows correspond to independent replicate foci. The 'uncorrected state' corresponds to a standard epifluorescence configuration (beam delivery angle of 0° and correction collar set to the coverslip thickness). The 'corrected' state refers to SlimVar including optimal settings of beam delivery angle (60° in water), telescope lens (32 mm towards objective) and correction collar (coverslip thickness - 30 µm). The 'overcorrected' measurement retains the oblique beam delivery angle and standard tube lens position, but the correction collar is adjusted by an equivalent amount in the opposite direction (coverslip thickness +30 µm) to indicate the impact of the spherical aberration removed in SlimVar. These examples *in planta* are of *FLC* foci identified in *z*-stacks (Fig. 7a) using a 'corrected' SlimVar configuration. (b) Lateral and (c) axial FWHM dimensions as determined from the PSF volume stacks (n = 2–4) using MetroloJ-QC (ImageJ plugin).[71] The axial FWHMs of beads prior to correction at zero depth agree qualitatively with the ideal diffraction-limited resolution[2] of $d_z = 0.89\,\lambda/\left(n - \sqrt{n^2 - NA^2}\right) =$ 380 nm. Axial FWHMs in the 'corrected state' at the calibration depth also agree with the empirical depth-of-field estimate of 530 nm (Supplementary Fig. 2a). The axial improvement demonstrated in beads is necessary and sufficient for feasible imaging at depths up to 30 µm inside plant tissue, in which the PSF is subject to additional aberration and scattering.

Source data are provided as a Source Data file.

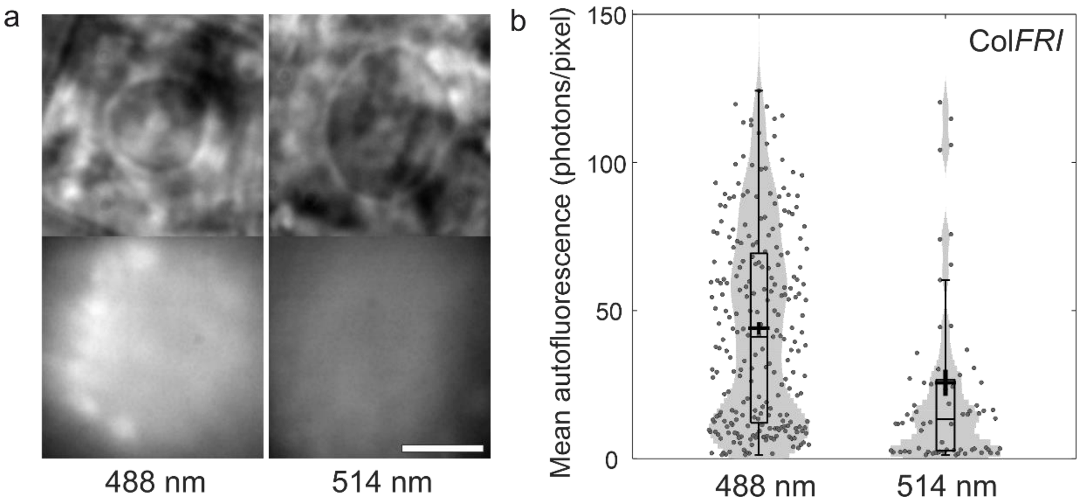

Supplementary Fig. 6. Autofluorescence controls for SlimVar microscopy.

a) SlimVar images of non-vernalised nuclei in the Col*FRI* wild-type (unlabelled) line; top, brightfield; bottom: SlimVar average projections over 100 frames, with contrast set to 120–370 counts (12–162 photons/pixel). Plastids and cell walls have higher autofluorescence than the nucleus, especially at 488 nm (bottom left), but do not contribute to false positives as they are removed during segmentation of the nucleus with the brightfield image. Scale bar: 5 µm. b) The mean number of background fluorescence photons per 53 nm pixel per 10 ms exposure in each nucleus for 488 nm and 514 nm excitation modes, aggregated over all vernalisation timepoints. The mean ± s.e.m. (s.d.) autofluorescence is 45 ± 4 (34) photons/pixel, or 26 ± 4 (33) photons/pixel under 488 nm and 514 nm (*n* = 250 and 71 nuclei) respectively. Source data are provided as a Source Data file.

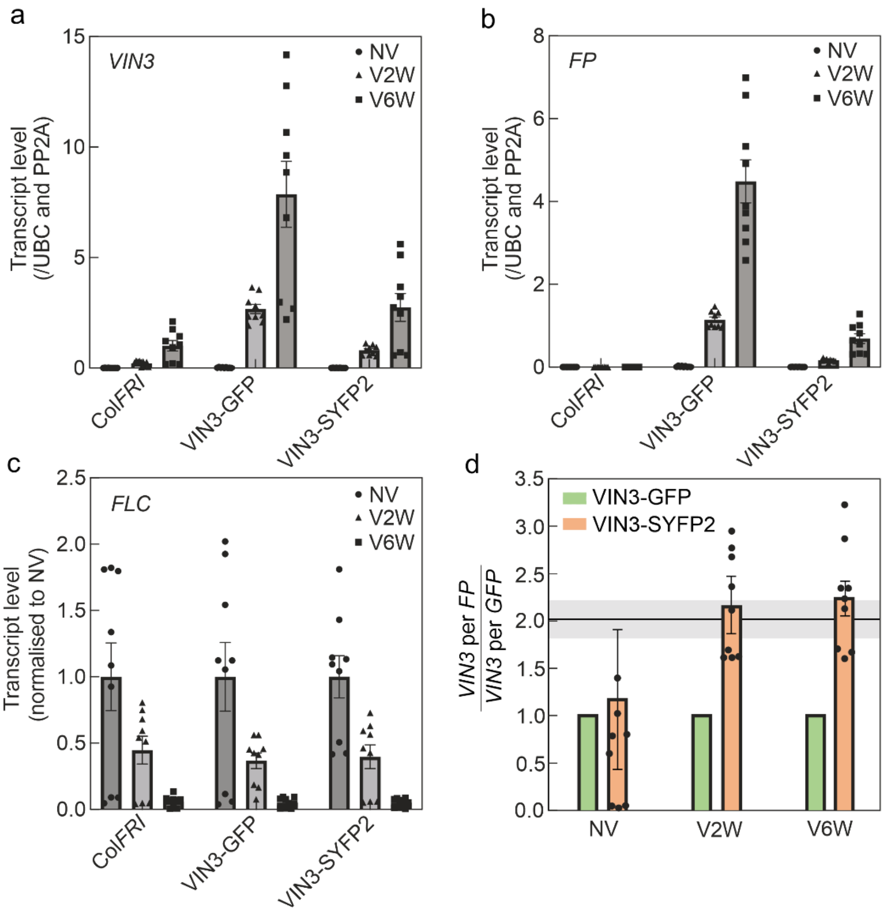

Supplementary Fig. 7. RNA expression analysis and characterization of VIN3-SYFP2.

Expression level of *VIN3* (a), fluorescent protein (b) in VIN3-GFP, VIN3-SYFP2 and Col*FRI* seedlings. (c) Expression level of spliced *FLC* in seedlings after 2 weeks (V2W) and 6 weeks vernalised (V6W) cold seedlings relative to non-vernalised (NV) seedlings. (a-c) Data are presented as mean ± s.e.m. ($N = 9$) from three biologically independent experiments. (d) Based on the assumption that protein levels scale proportionally with RNA levels, we derive a correction factor to infer the total number of VIN3 proteins in the yellow line from the detected number of VIN3-SYFP2 proteins. This factor can be estimated by taking the ratio of mRNA expression levels of *VIN3* to *SYFP2*, then normalising this by the ratio of *VIN3* to *GFP* expression in the green line, which lacks endogenous *VIN3*. The ratios for VIN3-GFP are therefore defined as 1. The error in the VIN3-SYFP2 correction factor is propagated directly from the s.e.m. of the independent measurements in panels a-b. Given the uniform rate of increase of VIN3 expression in all lines regardless of labelling, the correction factor is expected to be constant over time, albeit with a greater variance and background-related bias towards unity, at NV when expression is lowest. Taking an inverse-variance-weighted average over the three timepoints, the correction factor used in this work is 2.0 ± 0.2, which is consistent with the expected genotype of the yellow line: a single exogenous gene copy of *VIN3-SYFP2* and a single native gene copy of *VIN3*. Source data are provided as a Source Data file.

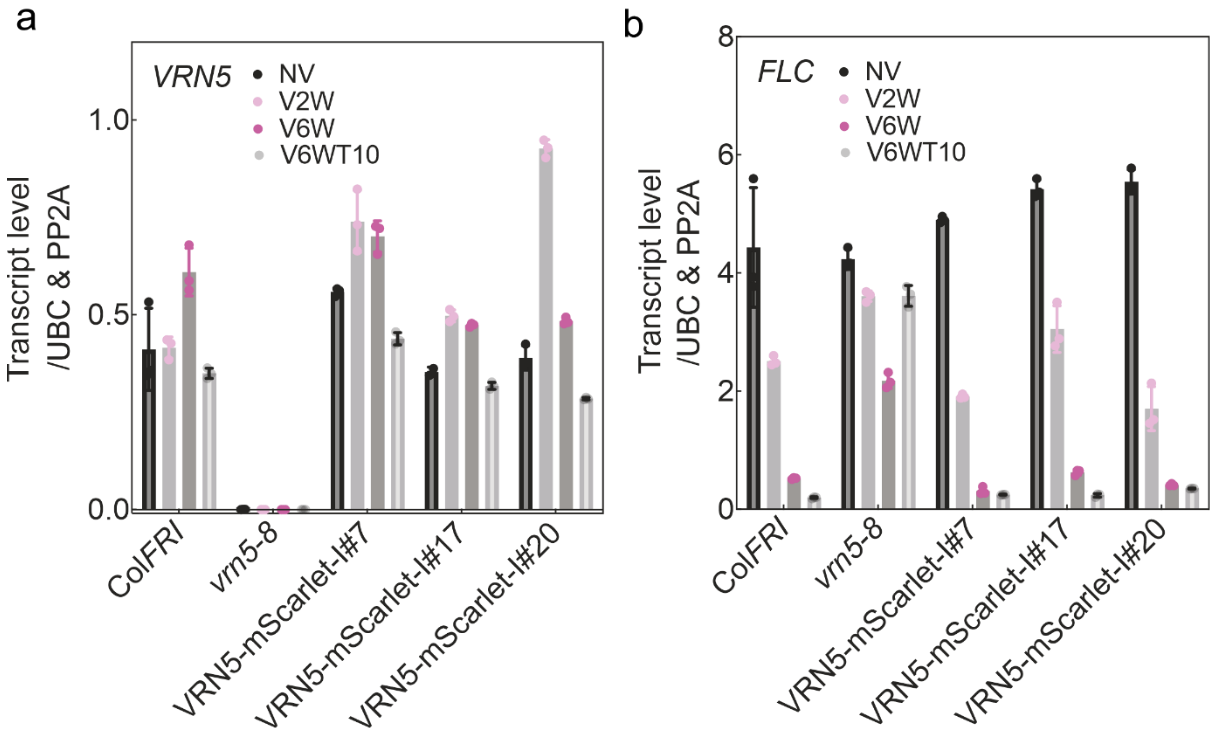

Supplementary Fig. 8. RNA expression analysis of *VRN5* and *FLC* in single-colour VRN5-mScarlet-I lines. Expression level of *VRN5* (a), of spliced *FLC* (b) in VRN5-mScarlet-I, *vrn5-8* and Col*FRI* seedlings before (NV) and after 2 weeks and 6 weeks cold treatments (V2W, V6W). V6T10 represents 10 days after a 6 week-cold treatment. Data are presented as mean± s.d. (N=3, except for the VRN5 data in VRN5-mScarlet-I#17, NV, where N=2) from 1 biological experiment. The samples of VRN5-mScarlet-I are from heterozygous T2 seedlings. Source data are provided as a Source Data file.

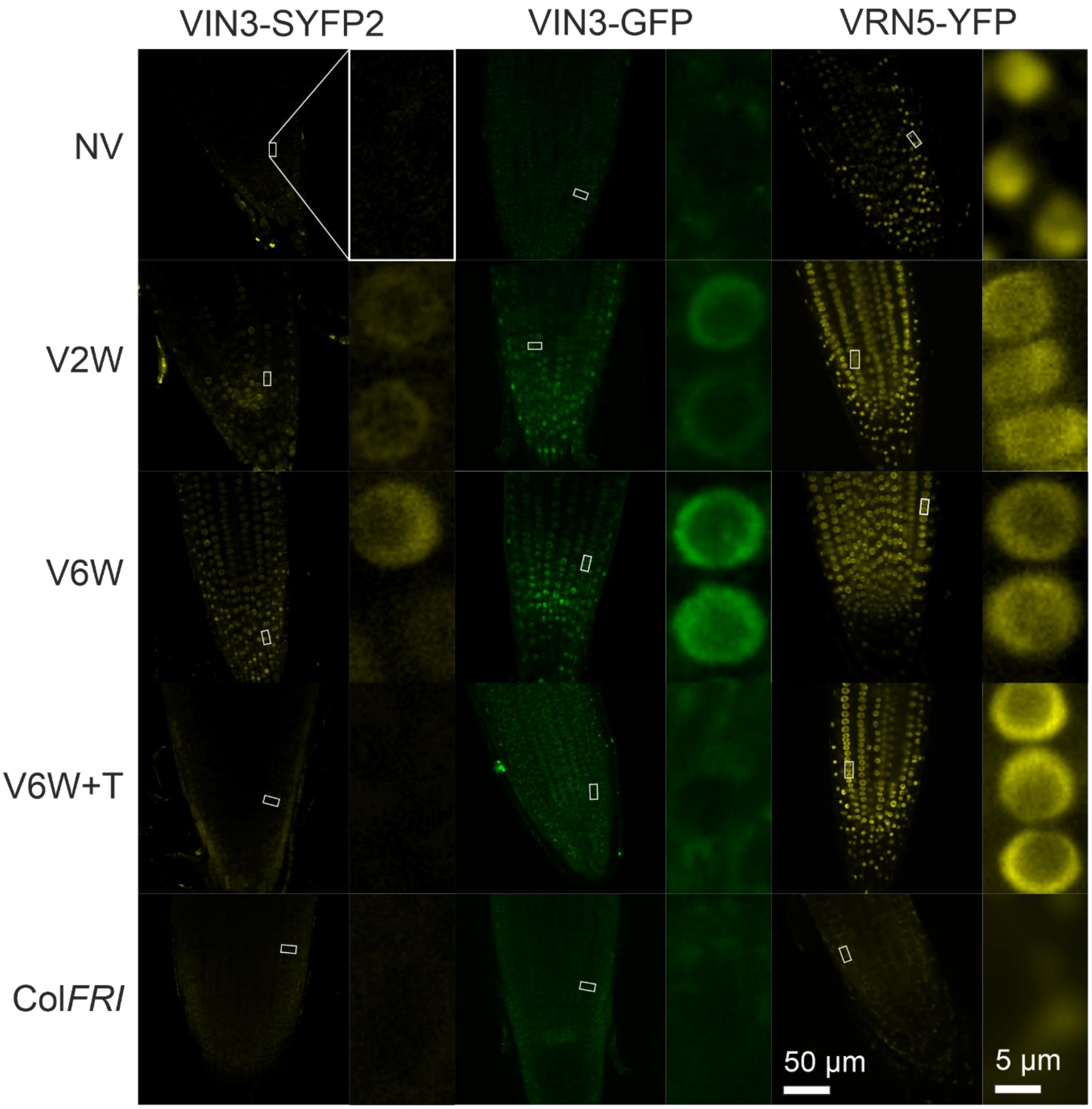

Supplementary Fig. 9. Confocal microscopy indicates VIN3 and VRN5 expression increase due to vernalisation.

Confocal microscopy projections of live whole root tips of VIN3-GFP and VRN5-YFP at different timepoints. NV is non-vernalised; V2W and V6W refers to 2 or 6 weeks of cold respectively; the post-vernalised timepoint V6W+T refers to 7 days warm (V6W+T7) for VIN3 and 14 days warm (V6W+T14) for VRN5. Bright particles outside the root envelope are remnants of the root cap. Also shown are insets at 13× scale to show detail of the labelled protein signals, which are consistently localised to the nucleoplasm, but not the nucleolus of each cell.

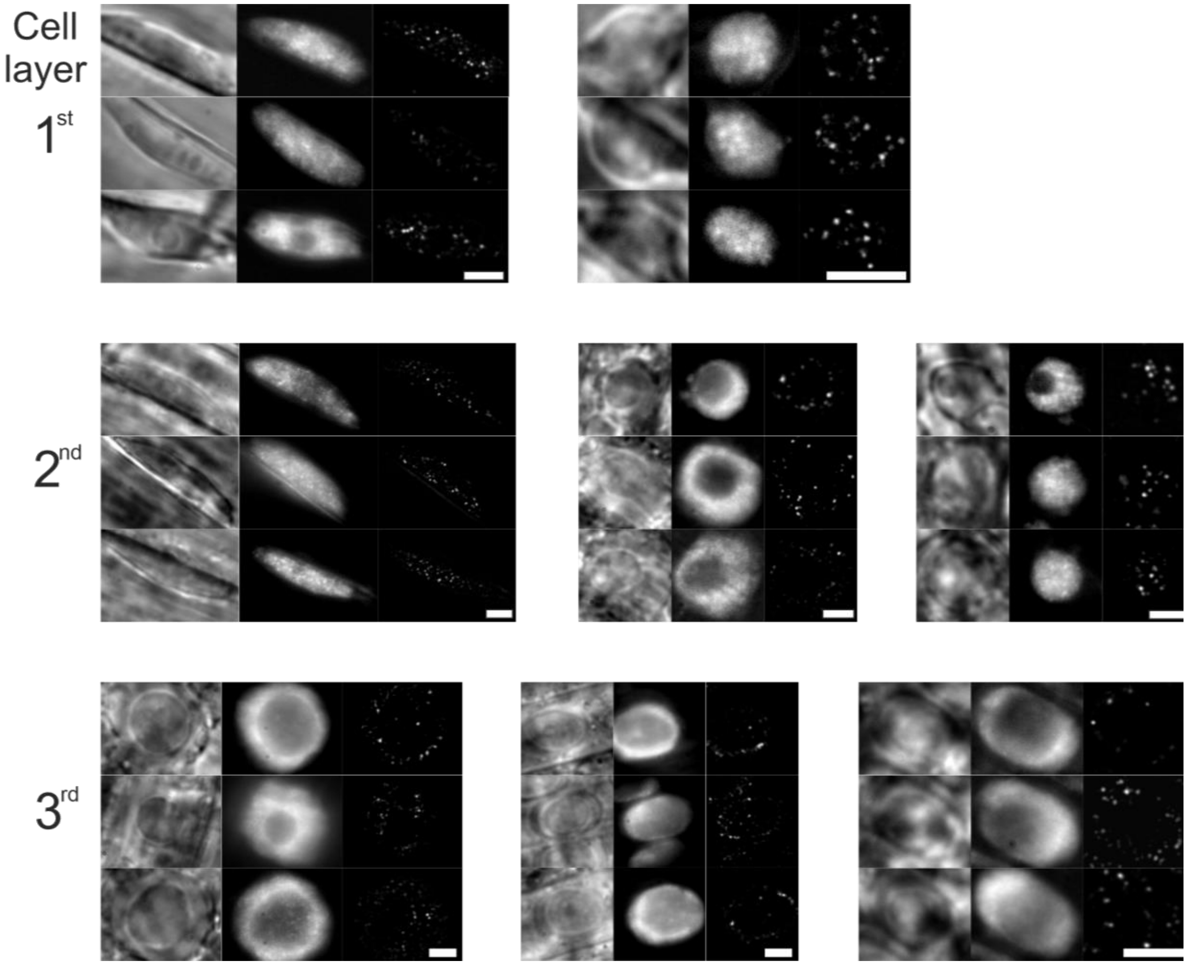

Supplementary Fig. 10. SlimVar detects molecular assemblies with high contrast across at least 3 cell layers in a range of cell morphologies.

SlimVar images of individual nuclei in the VRN5-YFP line, classified qualitatively by cell shape and location in root tip. Three independent fields of view (rows) are shown for each class at a mixture of timepoints before, during and after vernalisation. Left: brightfield, centre: initial fluorescence in SlimVar (average of first 3 frames); right: assemblies revealed by partial photobleaching during SlimVar sequence; visualisation generated in ImageJ from raw sequences by Gaussian smoothing at 1.0 pixel radius, gamma transform of 1.25, background subtraction at 10 pixel radius, followed by maximum projection over frames 30–100 in each sequence, then repeat background subtraction and Gaussian smoothing, then inverse gamma transform of 0.80. Exposure time: 10-20 ms. Scale bars: 10 μm.

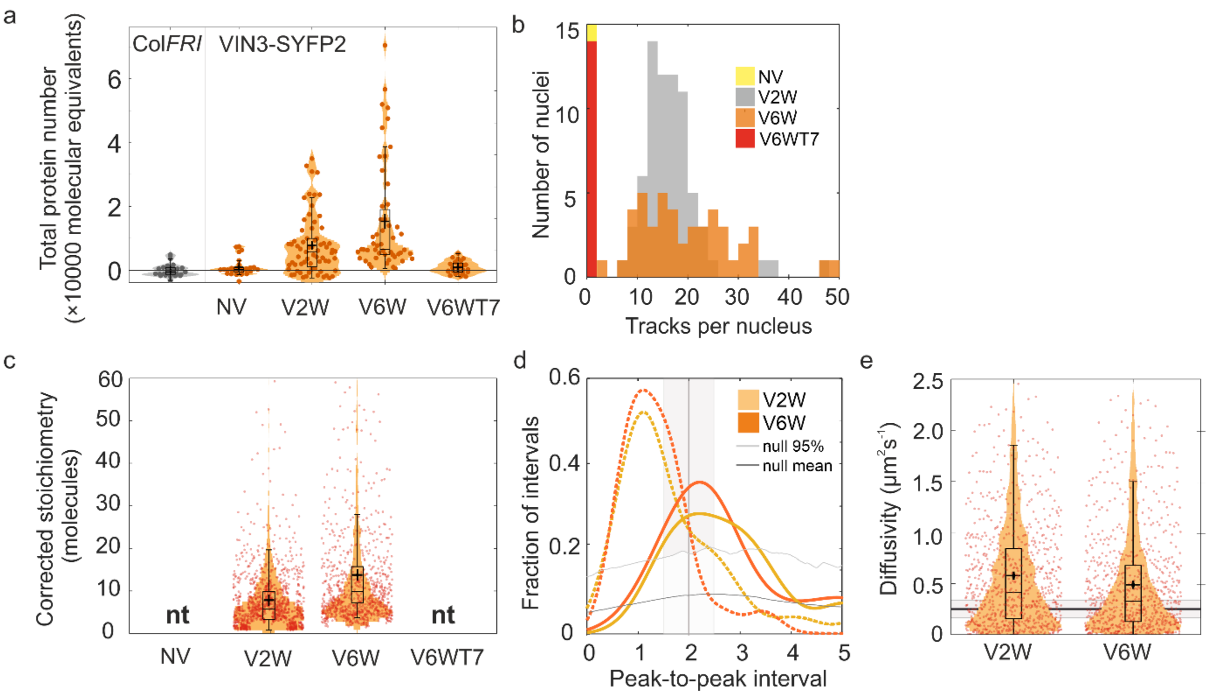

Supplementary Fig. 11. Collated SlimVar observations of VIN3-SYFP2.

a) Distributions of integrated nuclear intensity of VIN3-SYFP2 in individual nuclei before, during and after vernalisation. NV = not vernalised; V2W = two weeks of cold; V6W = six weeks of cold; V6WT7 = six weeks of cold followed by one week of warm conditions. In the VIN3-SYFP2 line, the unlabelled, endogenous fraction of VIN3 protein can be accounted for by a factor of 2.0 ± 0.2 derived from the relative mRNA expression levels (Supplementary Fig. 7). Bar, box and whisker denote median, interquartile range (IQR) and 1.5× IQR respectively; cross: mean ± s.e.m. Total protein numbers of VIN3-SYFP2 range from negligible before cold (410 ± 560 molecules, BM test vs. Col*FRI*, $N = 52$, $p = 0.52$), but reach 7,800 ± 1,600 molecules above background at 2 weeks cold ($N = 68$, $p = 2 \times 10^{-12}$) and rise further to 15,000 ± 3,000 molecules at 6 weeks cold ($N = 51$, $p = 0.0045$ vs V2W). At 7 days of warm, negligibly few VIN3-SYFP2 remain (900 ± 700 molecules per nucleus, BM test vs. Col*FRI*, $N = 54$, $p = 0.08$). b) The mean number of tracks is indistinguishable between VIN3-SYFP2 and VIN3-GFP, and between 2 and 6 weeks of vernalisation (V2W: 16.3 ± 1.0 and 18.3 ± 1.9, BM test, $N = 120$, $p = 0.42$; V6W: 17.4 ± 2.4 and 17.9 ± 1.8, $N = 140$, $p = 0.22$ respectively). c) After accounting for unlabelled VIN3, the mean labelled stoichiometry increases between 2 and 6 weeks' cold from 7.8 ± 2.7 to 13.8 ± 3.9 (BM test, $N = 1792$ tracks, $p = 3 \times 10^{-122}$). d) The stoichiometry distribution for VIN3-SYFP2 has a smaller periodicity (dashed lines) incompatible with an integer number of molecules greater than one. After accounting for unlabelled VIN3, periodicity (2.2 ± 0.8, V2W; 2.2 ± 0.8, V6W, solid lines) is consistent with dimeric subunits within assemblies during vernalisation. e) Diffusivity of VIN3 assemblies decreases during vernalisation (VIN3-SYFP2: $D = 0.58 \pm 0.02$ to 0.49 ± 0.02 μm^{2}s^{-1}; $N = 1,158$, $p = 0.00061$). Source data are provided as a Source Data file.

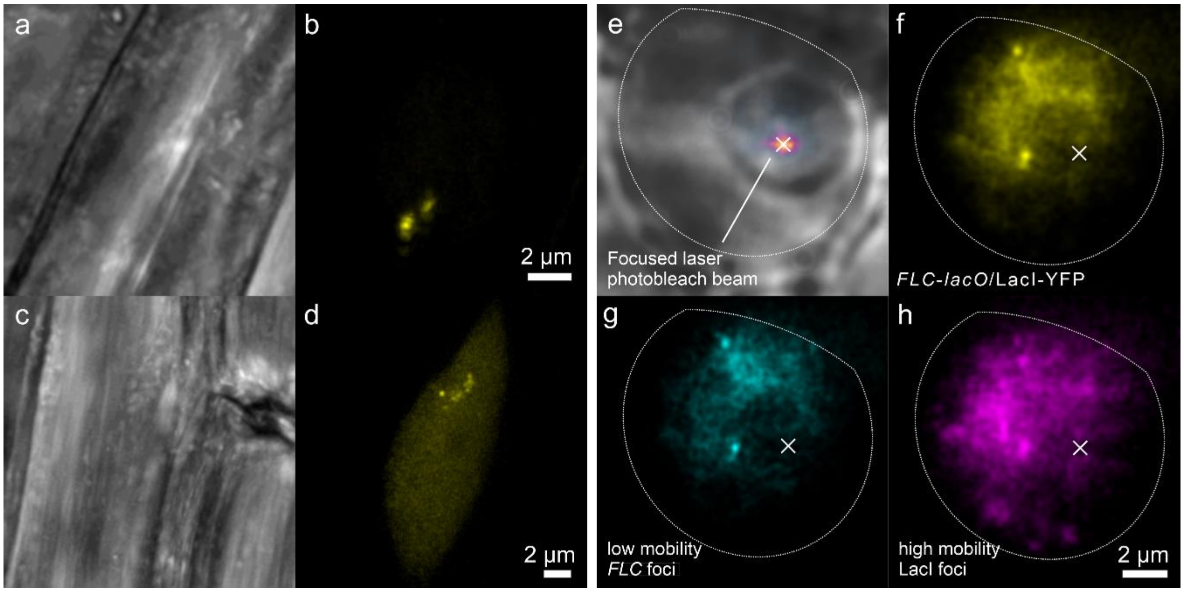

Supplementary Fig. 12. Confocal and SlimVar imaging of the FLC-*lacO*/LacI-YFP line. a-d) Confocal: a) Transmission and b) confocal fluorescence imaging of an non-vernalised nucleus in the epidermal meristem showing at least two loci, and c,d) a vernalised (six weeks of cold) nucleus in the epidermal elongation zone, featuring additional clustered pairs of *FLC* foci consistent with endoreduplication. Mobile LacI-YFP form a uniform background due to motion blur. Scale bars 2 µm. e-h) SlimVar: e) a large nucleus (dotted outline) of a non-vernalised cortical cell in brightfield with superposed intensity profile of a focused laser (cross) inside the nucleolus; an initial exposure with this beam, typically 30 s, removes excess unbound LacI-YFP that diffuses through the excitation volume, while preserving any bound to the *lacO* array at the *FLC* transgene; f-h) the subsequent SlimVar acquisition shown as f) mean average, g) median projection showing isolated and low mobility *FLC* foci at the expected frequency of ≥2 loci per nucleus, whose diffusivity is quantified and used to match the diffusivity of VIN3 and VRN5 under the same acquisition conditions (Supplementary Table 2); h) standard deviation projection indicating residual fast-moving LacI-YFP. In spot-bleached nuclei, we find a mean of 4.4 ± 0.8 slow LacI-YFP tracks per nucleus ($N = 38$ nuclei) reflecting the expected two *FLC* transgenes plus a minority of endoreduplication loci. At the observed particle number density, the likelihood of random overlap of >2 free LacI-YFP tetramers is negligibly low. We therefore identify all LacI-YFP tracks with stoichiometry >3 tetramers, or a threshold of 12 LacI-YFP, as potential *FLC* loci. These tracks exhibit diffusivity (Fig. 6a) of $D_{FLC} = 0.20 \pm 0.02$ µm^{2}s^{-1} ($N = 129$, mean ± sem, 20 ms exposure). Tracks in the upper quartile of LacI-YFP stoichiometry (>81 LacI-YFP molecules), corresponding to the brightest foci per nucleus, yield an equivalent diffusivity estimate of 0.17 ± 0.04 µm^{2}s^{-1} ($N = 36$; $p = 0.097$). With or without pre-bleaching, tracks with <12 LacI-YFP molecules typically diffuse faster, around 0.54 ± 0.04 µm^{2}s^{-1} ($N = 153$; $p = 0.0094$), which suggests they may consist of free LacI tetramers.

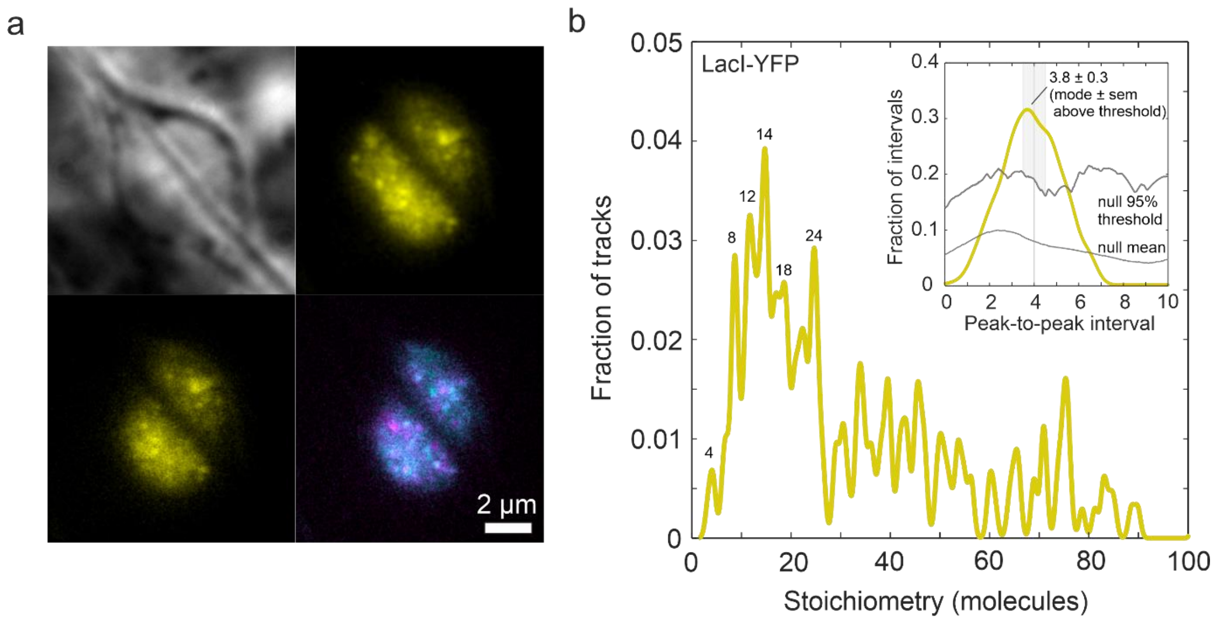

Supplementary Fig. 13. Tetrameric spacing of LacI-YFP validates peak interval extraction from stoichiometry distributions. a) Representative SlimVar images of neighbouring epidermal nuclei before vernalisation in single-colour *FLC-lacO*/LacI-YFP line under strong EtOH induction, shown as brightfield, initial SlimVar frame, and projections of mean (yellow), and merged median (cyan) and standard deviation (magenta) respectively over 5 frames. b) The stoichiometry of detected LacI-YFP tracks ($N = 225$) shows clear peaks at approximate multiples of 4, with occasional interceding multiples of 2, consistent with the tightly bound tetrameric (dimer-of-dimer) complex of LacI. The maximum stoichiometry observed is 380 molecules (not shown for clarity). Inset: the most common spacing between neighbouring peaks in the stoichiometry distribution as estimated by the new method. The threshold above which the null distribution can be rejected is the 95th percentile fraction of intervals (grey trace) output from simulated random stoichiometry (Methods). The most common interval is given by the modal peak value above the null threshold (3.8 ± 0.3 molecules), which is consistent only with integer 4 molecules, and thus a tetrameric repeat unit as expected. Source data are provided as a Source Data file.

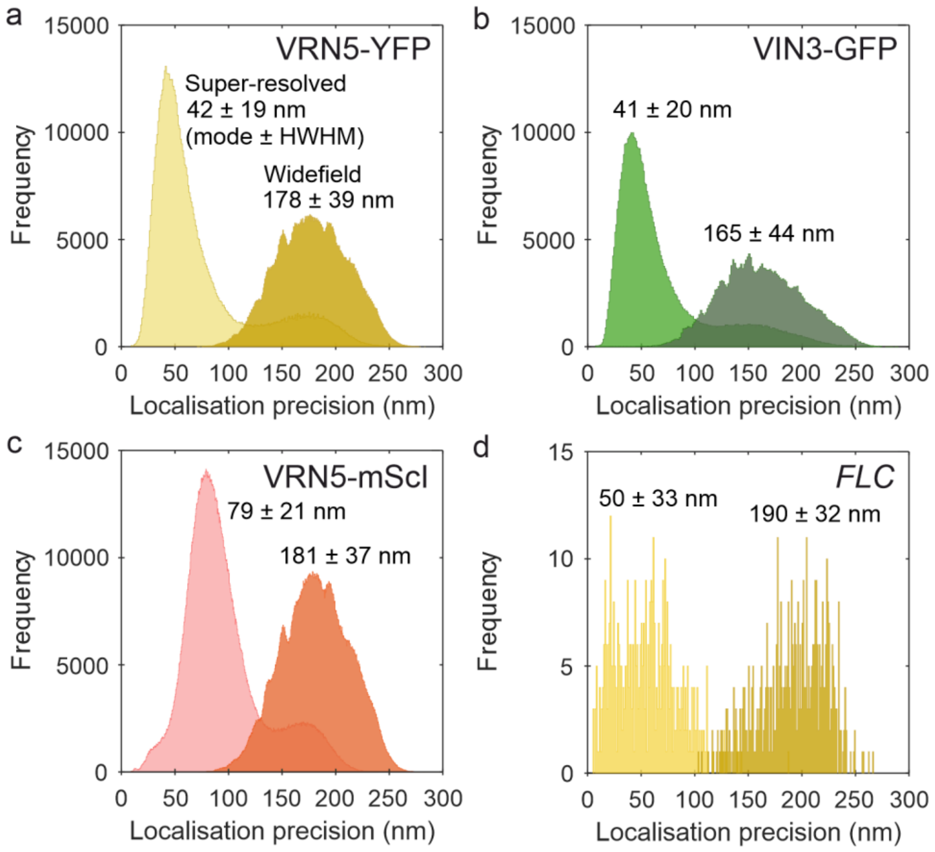

Supplementary Fig. 14. Localisation precision of tracked fluorescent foci in SlimVar acquisitions. Gaussian-fit widefield FWHM (major axis) and the corresponding super-resolved localisation precision estimated using the formulation of Thompson et al. [72] for foci within a) VRN5-YFP tracks ($N = 5326$). b) VIN3-GFP tracks ($N = 2479$) at 2 ms exposure per frame; c) VRN5-mScI tracks ($N = 6395$) and d) *FLC-lacO*/LacI-YFP loci ($N = 475$) at 20 ms exposure. The localisation precision becomes finer with greater photon counts in each foci. However, most localisations occur during later stages of photobleaching; these are relatively dim compared to background counts, and therefore do not reach the theoretical limit scaling as the inverse square root of the photon count. For the brightest foci, we observe values smaller than the lateral spatial localisation precision for a single molecule (40 nm) but these should be interpreted with caution since the physical size of each assembly is unknown. Source data are provided as a Source Data file.
