# Supplementary material for: SlimVar for rapid in vivo single-molecule tracking of chromatin regulators in plants
Source: Nat Commun. 2025 Sep 1;16:8156. doi: 10.1038/s41467-025-63108-8 (PMC12402101; doi:10.1038/s41467-025-63108-8)
Supplement: Supplementary file 3 — Description of Additional Supplementary Files [file 41467_2025_63108_MOESM3_ESM.pdf]

Description of Additional Supplementary Files

File Name: Supplementary Movie 1
Description: **SlimVar resolves dynamic VRN5-YFP assemblies in live root tips.**
A single nuclear acquisition of VRN5-YFP line in an epidermal cell (10 µm deep) at non-vernalised (NV) conditions showing distinct assemblies. Left: raw data; right: photobleach corrected and Gaussian smoothed by 1 pixel width to emphasise dynamic assemblies. Scale bar: 1 µm, frame duration 2 ms, frame rate 25 fps.

File Name: Supplementary Movie 2
Description: **VRN5-YFP assemblies in live root tips after vernalisation.**
A SlimVar acquisition in a large cortical nucleus (25 µm deep) in the VRN5-YFP line at post-vernalised (V6WT14) conditions showing large assemblies. Left: raw data; right: photobleach corrected and Gaussian smoothed by 1 pixel width. Scale bar: 2 µm, frame duration 20 ms, frame rate 10 fps.

File Name: Supplementary Movie 3
Description: **VIN3-GFP assemblies during vernalisation.**
A representative SlimVar acquisition of a smaller epidermal nucleus in VIN3-GFP line at 2 weeks' cold. VIN3 has significantly lower copy number and stoichiometry compared to VRN5, and background is higher in GFP channel relative to YFP. Scale bar 1 µm, frame duration 2 ms, frame rate 25 fps, as for Supplementary Movie 1.
