# Supplementary material for: SlimVar for rapid in vivo single-molecule tracking of chromatin regulators in plants
Source: Nat Commun. 2025 Sep 1;16:8156. doi: 10.1038/s41467-025-63108-8 (PMC12402101; doi:10.1038/s41467-025-63108-8)
Supplement: Supplementary file 7 — Reporting Summary [file 41467_2025_63108_MOESM7_ESM.pdf]

nature portfolio

Corresponding author(s): Mark C. Leake

Last updated by author(s): 31/07/2025

Reporting Summary

Nature Portfolio wishes to improve the reproducibility of the work that we publish. This form provides structure for consistency and transparency in reporting. For further information on Nature Portfolio policies, see our Editorial Policies and the Editorial Policy Checklist.

Statistics

For all statistical analyses, confirm that the following items are present in the figure legend, table legend, main text, or Methods section.

n/a	Confirmed	
☐	☒	The exact sample size (*n*) for each experimental group/condition, given as a discrete number and unit of measurement
☐	☒	A statement on whether measurements were taken from distinct samples or whether the same sample was measured repeatedly
☐	☒	The statistical test(s) used AND whether they are one- or two-sided<br>*Only common tests should be described solely by name; describe more complex techniques in the Methods section.*
☐	☒	A description of all covariates tested
☐	☒	A description of any assumptions or corrections, such as tests of normality and adjustment for multiple comparisons
☐	☒	A full description of the statistical parameters including central tendency (e.g. means) or other basic estimates (e.g. regression coefficient) AND variation (e.g. standard deviation) or associated estimates of uncertainty (e.g. confidence intervals)
☐	☒	For null hypothesis testing, the test statistic (e.g. *F*, *t*, *r*) with confidence intervals, effect sizes, degrees of freedom and *P* value noted<br>*Give P values as exact values whenever suitable.*
☒	☐	For Bayesian analysis, information on the choice of priors and Markov chain Monte Carlo settings
☒	☐	For hierarchical and complex designs, identification of the appropriate level for tests and full reporting of outcomes
☒	☐	Estimates of effect sizes (e.g. Cohen's *d*, Pearson's *r*), indicating how they were calculated

Our web collection on statistics for biologists contains articles on many of the points above.

Software and code

Policy information about availability of computer code

Data collection	Confocal and Airyscan images were acquired using ZEISS Zen Black v2.3.<br>SlimVar images were acquired with µManager v1.42: https://micro-manager.org/<br>All raw imaging data are available at the BioImage Archive: https://doi.org/10.6019/S-BIAD1217
Data analysis	Confocal and Airyscan images were postprocessed using ZEISS Zen Black v2.3 and FIJI/ImageJ2 v.1.54j.<br>SlimVar images were postprocessed using FIJI/ImageJ2 v.1.54j and ADEMScode v.2.2 in MATLAB v.R2023a for tracking analysis, available with documentation at https://github.com/alex-payne-dwyer/single-molecule-tools-alpd. All postprocessed images, analysed tracks and quantified properties are available at the BioImage Archive: https://doi.org/10.6019/S-BIAD1217.

For manuscripts utilizing custom algorithms or software that are central to the research but not yet described in published literature, software must be made available to editors and reviewers. We strongly encourage code deposition in a community repository (e.g. GitHub). See the Nature Portfolio guidelines for submitting code & software for further information.

Data

Policy information about availability of data

All manuscripts must include a data availability statement. This statement should provide the following information, where applicable:

- Accession codes, unique identifiers, or web links for publicly available datasets
- A description of any restrictions on data availability
- For clinical datasets or third party data, please ensure that the statement adheres to our policy

All raw and processed imaging data and analysed tracks are available at the BioImage Archive: https://doi.org/10.6019/S-BIAD1217. The ADEMScode MATLAB tracking analysis software can be found at https://github.com/alex-payne-dwyer/single-molecule-tools-alpd.

Research involving human participants, their data, or biological material

Policy information about studies with human participants or human data. See also policy information about sex, gender (identity/presentation), and sexual orientation and race, ethnicity and racism.

Reporting on sex and gender	This study did not use human participants or human data.
Reporting on race, ethnicity, or other socially relevant groupings	This study did not use human participants or human data.
Population characteristics	This study did not use human participants or human data.
Recruitment	This study did not use human participants or human data.
Ethics oversight	This study did not use human participants or human data.

Note that full information on the approval of the study protocol must also be provided in the manuscript.

Field-specific reporting

Please select the one below that is the best fit for your research. If you are not sure, read the appropriate sections before making your selection.

☒ Life sciences ☐ Behavioural & social sciences ☐ Ecological, evolutionary & environmental sciences

For a reference copy of the document with all sections, see nature.com/documents/nr-reporting-summary-flat.pdf

Life sciences study design

All studies must disclose on these points even when the disclosure is negative.

Sample size	We predetermined a target sample size of >24 cells total per line per condition, sufficiently powered to detect changes of <1 s.d. in each of the five test variables (number of tracks, nuclear protein copy number, stoichiometry, periodicity, diffusivity) at a Bonferroni-adjusted significance level of $\alpha = 0.05/5 = 0.01$. In the dual line, >10 cells per condition sufficed for estimates of track number and stoichiometry disaggregated by FLC colocalization.
Data exclusions	For single-colour experiments, no acquired data were excluded from analysis. For dual-colour colocalization experiments, acquisitions were retained for analysis only when meeting the prior condition that lacI-YFP foci consistent with FLC were satisfactorily observed under ethanol induction, as described in Methods.
Replication	We performed biological replicates, typically of >3 independent cycles of growth and vernalization, with >3 roots per cycle and >3 cells per root. Technical replicates were identified with tracks detected within each nuclear segment. Actual numbers of replicates analysed in each line and condition are detailed in Supplementary Table 2.
Randomization	Growth plates of seedlings were randomly assigned a vernalization timepoint. Within each replicate for a given line and condition, seedlings and cells were imaged in an arbitrary order towards the target sample size.
Blinding	Investigators were not blinded, since experimental protocol, and to a lesser extent analysis, necessarily varied by line genotype and vernalization timepoint.

Reporting for specific materials, systems and methods

We require information from authors about some types of materials, experimental systems and methods used in many studies. Here, indicate whether each material, system or method listed is relevant to your study. If you are not sure if a list item applies to your research, read the appropriate section before selecting a response.

Materials & experimental systems

n/a	Involved in the study
☒	☐ Antibodies
☒	☐ Eukaryotic cell lines
☒	☐ Palaeontology and archaeology
☒	☐ Animals and other organisms
☒	☐ Clinical data
☒	☐ Dual use research of concern
☐	☒ Plants

Methods

n/a	Involved in the study
☒	☐ ChIP-seq
☒	☐ Flow cytometry
☒	☐ MRI-based neuroimaging

Plants

Seed stocks

Seeds of published lines (VIN3-GFP [Yang et al. ref 8], VRN5-YFP [Greb et al. ref 16], FLC-lacO/lacI-YFP [Rosa et al. ref 53]) were obtained by Caroline Dean's group from their original stock at John Innes Centre.

Novel plant genotypes

VIN3-SYFP2/VRN5-mScarletI x FLC lacO/LacI-YFP: Mega-primer cloning used for tag exchange:GFP of pENTR pVIN3::VIN3-GFP [Qüesta et al, ref 70.] replaced by SYFP2; SYFP2 of pENTR pVRN5::VRN5-SYFP2 [Jones et al., ref 71] replaced by mScarlet-I, cloned to SLJ destination vectors (SLJ755I5/SLJ6991), transformed to Agrobacterium tumefaciens C58 by triparental mating. Floral dipping used to generate VIN3-SYFP2 in ColFRI background, and VRN5-mScarlet-I in vrn5-8 FRI mutant background. VRN5-mScarlet-I crossed into FLC-lacO/lacI-YFP line, of which imaging experiments used T1 generation seedlings selected by multiple antibiotic growth media.

Authentication

In addition to antibiotic selection, copy number was determined in T1/T2 transformants by IDna Genetics (Norwich Research Park).
